# Effect of Nickel-Chromium and Non-Precious Gold Color Alloy Cast Posts on Fracture Resistance of Endodontically Treated Teeth 

**DOI:** 10.22037/iej.v12i3.10586

**Published:** 2017

**Authors:** Fatemeh Gholami, Parisa Kohani, Shima Aalaei

**Affiliations:** a *Dental Caries Prevention Research Center, Qazvin University of Medical Sciences, Qazvin, Iran;*; b *Private Practice, Qazvin, Iran*

**Keywords:** Alloys, Casts, Fracture, Nickel-Chromium, Resistance

## Abstract

**Introduction:**

Root canal-treated teeth are weaker than vital teeth and are more susceptible to fractures. Therefore, special precautions should be adhered to, such as the use of various types of cast or prefabricated posts. Regarding the effect of post material on fracture resistance of teeth, the aim of this *in vitro* study was to evaluate the effect of non-precious gold color alloy (NPG) and nickel-chrome (Ni-Cr) cast posts on resistance of endodontically treated teeth.

**Methods and Materials:**

In this study, 30 freshly extracted single-rooted premolar teeth were randomly divided into two groups. After root canal treatment, post patterns were made with Duralay in group 1 and cast with Ni-Cr alloy; in group 2, the patterns were cast with NPG alloy. Zinc phosphate cement was used for cementation in this study. Shear force was applied at 1 mm/min at 45^º^C to the buccal cusps until root fracture occurred. Independent sample *t* test was used for data analysis by using SPSS version 21. The level of significance was set at 0.05.

**Results:**

Mean fracture resistance values were 1380±454 N for Ni-Cr versus 1964±640 N for NPG, with significant differences (*P*=0.007).

**Conclusion:**

The fracture resistance of endodontically treated teeth with non-precious gold color alloy cast post was higher than that of endodontically treated teeth with Ni-Cr cast post.

## Introduction

Non-vital teeth usually exhibit biomechanical defects such as insufficient remaining tooth structure due to caries or previous restorations [1]. These teeth usually have poorer physical properties compared to vital teeth due to changes in crosswise collagen connections, destruction of the nerve reaction mechanism, a decrease in moisture, a decrease in the remaining tooth structure and stresses produced during post placement procedures or during function and are prone to fracture [2]. When the crown structure is lost completely, the retention of the core is provided from the root canal space, by placement of a post [3]. The principal function of a post is to provide sufficient retention for the core and crown from the root canal space of the tooth. In addition, the post helps in distribution of the tensions more evenly in tooth structure by distributing the functional forces on a larger surface area of the remaining root [4, 5].

After carrying out extensive research since 1990s, researchers have concluded that the difference between the modulus of elasticity of dental materials and that of tooth dentin is an important factor in transferring functional forces. The difference in the modulus of elasticity of these materials might result in discrepancies in distribution of tensions in tooth roots [6, 7]. Prefabricated metallic posts have sufficient strength but they pose many problems due to corrosion, such as difficulty in removing the post from the root canal space in case of the need for endodontic retreatment, high modulus of elasticity in comparison to dentin and an increase in the odds of crack formation and un-restorable vertical fractures in the root [8, 9]. Ni-Cr alloy is routinely used for cast posts [6, 7]. In recent years, a non-precious gold color alloy (NPG) has been introduced, which has surface characteristics and applications similar to yellow gold alloys but with a lower cost. This alloy exhibits easy casting properties, with accurate and high-quality adaptation. Some of the properties of this alloy are high durability, high mechanical and thermal strength, excellent fit, easy soldering and finishing, and biocompatibility. It is not clear at present which material or restorative technique is more durable and more appropriate [10].

The present study was undertaken to determine the effects of NPG and Ni-Cr alloys posts on the fracture resistance of endodontically treated teeth, considering a paucity of data on the effect of NPG alloy on the fracture resistance of tooth roots.

## Materials and Methods

Thirty freshly extracted single-rooted premolars, with almost similar dimensions, were included in the present *in vitro* study. The samples measured at least 13 mm in root length, from the apex to the CEJ, had no cracks, previous root canal therapies, and their restorations were 2 mm above the CEJ. The tooth crowns were removed at the CEJ level on the buccal aspect and then the samples were stored in normal saline solution. The samples underwent root canal treatment using the step-back technique. The root canals were cleaned and shaped up to #35 as the master apical file and irrigated with 5.25% NaOCl. Then the root canals were obturated using the lateral condensation technique. The root canal orifices were covered with Cavit temporary restorative material (ESPE-Premier, Norristown, PA, USA), followed by storage of the samples in normal saline solution for 24 h. To simulate the periodontal ligament, the samples were embedded in a thin silicone layer (Plasta-Dip, Blaine, MN, USA), with a homogeneous thickness of 0.1-0.2 mm on the root surface, 2 mm apical to the CEJ (location of the cut). After placement of the samples in autoplymerized acrylic resin blocks, Cavit was removed and the post space was prepared at a length of 10 mm using Peeso reamer drills up to #4 (Mani, Tochigi, Japan). The cast post pattern was fabricated on each sample with the direct technique, using Duralay acrylic resin (Inlay Pattern Resin Duralay, Reliance Dental MFG Co, Worth, IL, USA), followed by fabrication of the acrylic core, resembling a prepared tooth with a buccal and lingual cusp. After preparation, the samples were randomly divided into two groups (*n*=15). In groups 1 and 2, Ni-Cr and NPG alloy posts were used, respectively. After cementation of the cast posts and cores with zinc phosphate cement, they were held in place for 30 sec, using finger pressure. Excess cement was removed from the tooth surface after cement setting. Then all the samples were stored in normal saline solution at 37^°^C for 24 h. After preparation of the teeth, a universal testing machine (Zwick/Roell Z 020, Ulm, Germany) was used to apply a shearing force to the buccal cusp of the core at a crosshead speed of 1 mm/min at an angle of 45^°^C until fracture occurred. This force was recorded in data sheets.

Descriptive statistical methods were used to calculate means and standard deviations of the force necessary to induce fracture. Independent *t* test was used to compare the means between the two groups. Data were analyzed with SPSS software (SPSS, version 21.0, Chicago, IL, USA).

## Results

In the present *in vitro* study, 15 samples in the Ni-Cr group and 15 samples in the NPG group were evaluated. The mean fracture forces in the Ni-Cr and NPG groups were 1380±454 and 1964±640 N, respectively (Table 1). 

Comparison of the mean root fracture forces between the two groups with independent *t* test showed higher fracture resistance in the NPG group compared to the Ni-Cr group and the mean difference between the two groups was significant (*P*=0.007) (Table 2).

## Discussion

Researchers and clinicians are always making efforts to render treatment and transfer functional forces properly in association with provision of esthetic appearance and tooth strength. Normally, if the lost tooth structure makes it impossible to gain adequate retention for the restoration, it is necessary to place a post to function as a replacement for the lost tooth structure and provide retention for the restoration. The post should be meticulously selected to meet the retention needs of the tooth.

**Table1.** The statistical indices in the study groups

**Group (N=15)**	**Mean (SD)**	**Min**	**Max**
**Ni-Cr **	1380 (454)	400	1980
**NPG **	1964 (640)	630	3300

**Table 2:** Comparison of the mean fracture resistance values between the two groups using independent *t*-test

**Group (N)**	**Mean **	**Mean difference **	**T**	***P*** **-value**
**Ni-Cr (5)**	1380	584	2.88	0.007
**NPG (15)**	1964			

In other words, the tooth and the restoration should not be adapted to the post; rather a post system suitable for the condition of the tooth should be selected. When a post is prepared to provide retention for the core, the post space should be prepared minimally, in terms of length, diameter and convergence and based on the needs. Excessive preparation of the post space and excessive removal of dentin weaken the root, making it pone to fracture. In addition, the type of the alloy used for the fabrication of the post is effective in fracture resistance of the tooth root [11]. 

In the present study, the mean fracture resistance values in the Ni-Cr and NPG cast post groups were 1380±454 and 1964±640 N, respectively. The fracture resistance in the NPG group was higher than that in the Ni-Cr group and the difference was significant (*P*=0.007).

The strength of endodontically treated teeth with the use of NPG cast post was higher than those with the use of Ni-Cr cast post; indicating that NPG cast posts were more appropriate for the restoration of endodontically treated teeth and for decreasing the odds of tooth fracture.

Sabouhi *et al*. [12] compared fracture resistance of teeth restored with cast posts and cores with that of teeth restored with fiber glass posts and composite resin cores. Mahmoudi *et al.* [11] evaluated the dimensions and the parallel and conical geometric forms and ceramic, nickel-chromium and alumina posts, and Nakamura *et al.* [13] evaluated gold alloy cast posts and cores, stainless steel posts and resin cores and fiber posts and resin cones, all concluding that the material of the post used had a significant effect on fracture resistance of the root, which might be attributed to differences in the elements constituting these alloys. In addition, it was concluded that the type of the post used (cast or prefabricated) had a relatively significant effect on root fracture resistance. 

A study by Mezzomo *et al*. [14] showed that posts and cores fabricated with gold alloy exhibited higher fracture resistance compared to fiber glass posts with composite resin cores and resulted in lower stress in the root dentin. The studies above are consistent with the present study in that fracture resistance changes with a change in the type of the alloy used to fabricate the post, considering the fact that the alloys used in the posts were different. Another factor affecting the fracture resistance is the geometric shape of the post. In cast posts (similar to the present study) the post was conical in shape and these posts transfer stresses to different parts of the root and increase the fracture resistance of the root, which was shown by Mahmoudi *et al*. [11]. In the present study an attempt was made to eliminate the effect of post shape on fracture resistance by selecting identical posts in terms of diameter and shape. 

The fracture resistance of NPG cast posts in the present study (1965 N) was very high and such high force has not been recorded in many studies. For example, the highest fracture resistance in the study by Sabouhi *et al. *[12] was 1489 N for cast posts compared to fiber glass posts with composite resin cores (1195 N); in a study by Asadzadeh Aghdaei and Ghanbarzadeh [15] a fracture resistance of 407 N was recorded for Ni-Cr posts compared to 335 N for para post with a composite resin core and 371 N for FRC prefabricated post and composite resin core. Undoubtedly, the type of the alloy (NPG) was one of the most important factors involved in the high fracture resistance of the root recorded in the present study compared to other studies. Of course, the tooth type and the number of roots, too, can affect the fracture resistance. In the present study single-rooted premolars were used; however, in studies above, different tooth types have been used such as canine, central and molar teeth of the maxilla and mandible. Other factors responsible for differences between the results might be the type of the core and cement used and the force application technique during the fracture test.

In a study by Haghighi *et al.* [16] fracture resistance of endodontically treated teeth restored by NPG cast post/cores was significantly higher than that of those restored with nickel-chromium post/cores, consistent with the results of the present study. However, Khaledi *et al.* [17] found that the resistance fracture of non-vital teeth restored by nickel-chromium cast post/cores was significantly more than those restored by NPG cast post/cores, which was contrary to results of current study. The reason for this contrast could be that they studied on central incisors and cut crowns into horizontal sections at a line 2 mm incisal to the CEJ; they also cemented the posts with glass ionomer (GI); but the main difference was using indirect technique of post preparation in Khaledi’s article.

The adaptation of the post with root canal walls affect the fracture resistance of teeth [16]. Since in the present study two cast post/core systems were compared, the posts had the highest adaptation and the cement occupied the least space. Therefore the differences could be due to different elastic modulus of the casting alloys. Since the elastic modulus of Ni-Cr is higher than NPG, it was rigid when the force was applied and did not longer follow the elastic deformation, which resulted in localized stresses within the root canal, leading to root fracture [18]. 

## Conclusion

Based on the results of the present study, the fracture resistance of endodontically treated teeth restored with cast NPG post-and-cores was higher than those treated with nickel-chromium post-and-cores. Therefore NPG alloy is recommended for cast posts in endodontically treated teeth.
